# Do Obesity and Adipose Tissue Cytokines Influence the Response to Janus Kinase Inhibitors in Rheumatoid Arthritis?

**DOI:** 10.3390/nu17050820

**Published:** 2025-02-27

**Authors:** Marta Novella-Navarro, Ana Van Den Rym, Chary López-Pedrera, Ana Martínez-Feito, Beatriz Nieto-Carvalhal, Keren Reche, Clementina López-Medina, Alejandro Escudero-Contreras, Pilar Nozal, Maria Eugenia Miranda-Carús, Irene Monjo, Eugenio De Miguel, Alejandro Balsa, Rebeca Pérez-De Diego, Chamaida Plasencia-Rodríguez

**Affiliations:** 1 Rheumatology Department, Hospital Universitario La Paz, 28046 Madrid, Spain; mariaeugenia.miranda@salud.madrid.org (M.E.M.-C.); irenemonjo@gmail.com (I.M.); eugenio.miguel@salud.madrid.org (E.D.M.); al.balsa@ser.es (A.B.); chamiplaro@gmail.com (C.P.-R.); 2 Immuno-Rheumatology Investigation Group, IdiPAZ, Hospital Universitario La Paz, 28046 Madrid, Spain; bea.nc97@gmail.com; 3 Laboratory of Immunogenetics of Human Diseases, IdiPAZ, Hospital Universitario La Paz, 28046 Madrid, Spain; annevandenrym@gmail.com (A.V.D.R.); perezdediego@gmail.com (R.P.-D.D.); 4 Rheumatology Department, Hospital Reina Sofía, IMIBIC, University of Córdoba, 14071 Córdoba, Spain; charylopezpedrera@gmail.com (C.L.-P.);; 5 Immunology Department, Hospital Universitario La Paz, 28046 Madrid, Spain; ana.martinez.feito@gmail.com (A.M.-F.);; 6 Lymphocyte Pathophysiology in Immunodeficiencies Group, La Paz Institute for Health Research (IdiPAZ), 28046 Madrid, Spain; keren.reche.yebra@idipaz.es

**Keywords:** obesity, adipose tissue cytokines, targeted synthetic disease-modifying antirheumatic drugs, rheumatoid arthritis

## Abstract

**Background**: Obesity is a frequent comorbidity in rheumatoid arthritis (RA). This condition may lead these patients to have poorer disease control and a worse response to some of the available treatments. **Objectives**: We aim to analyze the role of body mass index (BMI) in the clinical response to Janus kinase inhibitors (JAKis) in patients with RA. We aim to perform an in-depth analysis of the pathophysiology of obesity by assessing serum adipokine levels, their potential influence in disease activity and their changes with treatment. **Methods**: This study involved 81 patients with RA treated with JAKis from Hospital La Paz and Hospital Reina Sofía. Patients were classified according to their BMI as normal weight and overweight/obesity. The clinical response to treatment was assessed by the Clinical Disease Activity Index (CDAI) and Disease Activity Score-28 (DAS28) 6 months after the initiation of JAKis. Serum adipokines (leptin and adiponectin) were determined using a commercial immunoassay kit in samples obtained before the initiation of JAKis and after 6 months of treatment. **Results**: Leptin levels showed a significant positive correlation with BMI at baseline (r = 0.59, *p* < 0.01) and at 6 months (r = 0.56, *p* < 0.01) in the whole cohort, but no correlation was found between BMI and adiponectin. No correlation between disease activity and BMI was found in the whole cohort at baseline and at 6 months measured by both the CDAI and DAS28. Fifty patients (61.7%) achieved low disease activity (LDA)/remission at 6 months, regardless their BMI, and no differences in serum adipokine levels were observed at baseline and at 6 months in patients who achieved LDA vs. no-LDA. **Conclusions**: In this study, we did not find an association between obesity and the extent of LDA in patients treated with JAKis; therefore, this mechanism of action could be suitable for overweight/obese patients with RA.

## 1. Background

In recent years, interest in the field of obesity has grown exponentially due to its increasing prevalence [1]. Obesity predisposes individuals to metabolic and cardiovascular diseases, but it has also been linked to chronic inflammatory diseases. This is because adipose tissue functions as an endocrine organ, playing an important role not only in metabolism regulation but also in immunological and inflammatory processes [2].

The progressive and excessive accumulation of fat that occurs in obesity leads to substantial changes in the quantity and phenotype of immune cells residing in adipose tissue. This results in an increase in both the number and activity of some immune cells, particularly macrophages, neutrophils, T lymphocytes (Th17), and B lymphocytes, while reducing others, such as eosinophils and certain subtypes of T lymphocytes (Th2 and Treg) [3]. Blood levels of macrophage apoptosis inhibitors (MAIs) increase under obesity conditions. First, they induce lipolysis, producing saturated fatty acids. These fatty acids, in turn, act on adipose tissue by promoting the infiltration of M1 macrophages and activating the NLRP3 inflammasome, which secretes IL-1β and IL-18—both involved in the pathogenesis of immune-mediated diseases (IMIDs). Secondly, the MAI forms immune complexes with natural autoreactive IgM associated with autoantigens, promoting their retention in follicular dendritic cells. The subsequent presentation of autoantigens to follicular B cells leads to the production of IgG autoantibodies [3,4].

Moreover, both adipocytes and immune cells that massively infiltrate adipose tissue secrete cytokines, which also contribute to the perpetuation of a pro-inflammatory state. Thus, adipose tissue can secrete and release cytokines such as TNFα and IL-6, which are common in inflammatory diseases like rheumatoid arthritis (RA), as well as cytokines known as adipokines (leptin, adiponectin, resistin, and visfatin, among others) [5]. Most of these have pro-inflammatory properties, contributing to an inflammatory state associated with obesity [5,6]. This connection between obesity and chronic inflammation supports the idea that adipokines may contribute to the pathogenesis of numerous inflammatory diseases, including rheumatic diseases.

The role of adipokines in the mechanisms of rheumatic diseases is supported by evidence that they can exert a modulatory effect on target tissues involved in these pathologies, such as synovial, cartilage, bone, and immune cells. Additionally, although adipokines are predominantly produced by adipose tissue, they may also be expressed in joints by chondrocytes, synoviocytes, and resident immune cells. Leptin is considered a pro-inflammatory adipokine that stimulates the production of other cytokines such as TNFα, IL-6, and reactive oxygen species, which in turn induce the production of chemokines and alter the Th1/Th2 cell profile [7]. This further increases leptin secretion and contributes to perpetuating the pro-inflammatory state. Advances in the study of this adipokine have led to its recognition as a key factor in the development of RA [7].

Adiponectin has traditionally been considered an anti-inflammatory adipokine, capable of regulating and acting as a protective factor in the development of metabolic diseases. Paradoxically, in the pathogenesis of RA, it appears to have a pro-inflammatory effect at the joint level by stimulating the secretion of mediators such as IL-6, TNFα, IL-8, and prostaglandin E2, among others. However, the role of adiponectin in RA remains controversial. Although higher levels of this adipokine have been detected in RA patients compared to healthy individuals, there are contradictory findings regarding its role in disease activity [8,9].

Due to all the aforementioned aspects regarding the role of obesity and adipokines in RA, there has been growing interest in evaluating how these factors influence the response to different treatments. So, this study aims to explore the influence of BMI on the clinical response to Janus kinase (JAK) inhibitors in RA patients. Additionally, it investigates the pathophysiology of obesity by measuring serum adipokine levels and assessing their potential role in treatment responses.

## 2. Methods

This study involved subjects with RA from the prospective cohort of patients drawn from the Rheumatoid Arthritis Registry at La Paz University Hospital and Hospital Reina Sofía between January 2017 and June 2024. Ethical approval was obtained from the La Paz Ethics Committee (PI-5608).

Inclusion criteria were as follows: RA patients aged ≥18 years according to the 1987 ACR or 2010 ACR/EULAR classification criteria with active disease and treated with JAKis (baricitinib, filgotinib, tofacitinib and upadacitinib) at standard doses. Patients with contraindications to start treatment with JAKis and patients with overlapping syndromes were excluded.

Patients were classified according to their BMI in normal weight (19–24.9 kg/m^2^) and overweight/obesity (≥25 kg/m^2^).

### 2.1. Data Collection

For all patients, the following data were collected just prior to starting JAKis: demographic characteristics (age, sex, BMI, and smoking habit), age at the diagnosis of RA, age at JAKi initiation, concomitant treatment with conventional synthetic csDMARDs and previous treatment with bDMARDs, laboratory parameters such as rheumatoid factor (RF) and anti-citrullinated peptide antibodies (ACPAs), C-reactive protein (CRP), and the erythrocyte sedimentation rate (ESR).

### 2.2. Assessment of Clinical Disease Activity and Response to Treatment

Disease Activity Score-28 (DAS28) and Clinical Disease Activity (CDAI) were assessed at baseline and 6 months after starting JAKi. At 6 months, patients were classified according to their response to treatment (assessed by DAS28 and the CDAI) in two groups: patients who achieved low disease activity (LDA) defined as DAS28 < 3.2 or CDAI ≤ 10 and patients who did not achieve this outcome (No-LDA) defined as DAS28 > 3.2 or CDAI > 10. The DAS28 was assessed due to its wide use in clinical practice and the CDAI to avoid the use of indexes containing acute phase reactants, given the effect JAKis on CRP.

### 2.3. Measurement of Serum Adipokines

Leptin and adiponectin serum levels were assessed at baseline and at 6 months of treatment initiation. Commercial immunoassay kits were used to measure human leptin and adiponectin in serum samples obtained at baseline and after 6 months of treatment (Human Leptin ELISA kit, EZHL-80SK and Human Adiponectin ELISA kit, EZHADP-61 K, Merck Millipore, S.A.S., Madrid, Spain), according to the manufacturer’s instructions. All samples were analyzed in duplicate and quantified relative to a standard curve, using a 5- and 4-parameter algorithm, respectively.

Statistical analysis: Descriptive analyses of all patients were performed. Data are reported as absolute numbers and frequencies for qualitative variables and for quantitative variables, median and interquartile range (IQR), or mean and standard deviation (SD), depending on the distribution of the data. The chi-square and Fisher exact tests were used to assess differences between qualitative features and for quantitative variables, along with T-Student, U-Mann–Whitney and Wilcoxon tests. Correlations between adipokines and other quantitative variables were assessed using Pearson and Spearman coefficients. *p*-values < 0.05 were considered statistically significant (IBM SPSS 21.0), and figures were created with GraphPad Prism 8.0.0.

## 3. Results

### 3.1. Baseline Clinical and Sociodemographic Characteristics

In total, 81 RA patients were included from Hospital La Paz and Hospital Reina Sofía starting JAKis. The most prescribed JAKi was baricitinib (45.7%) followed by filgotinib (21.0%), upadacitinib (17.3%) and tofacitinib (16.0%). We classified our population in two groups regarding BMI, and from the whole cohort, 44 patients (54.3%) had a BMI ≥ 25 kg/m^2^, and among these patients, 33 had a BMI of 25–29.9 kg/m^2^ (overweight); 5 had a BMI of 30–34.9 (grade I obesity) and 6 had a BMI of 35–39.9 (grade II obesity). No differences in DAS28 at JAKi initiation between patients with overweight/obesity and normal weight were found [(4.3 ± 1.0) vs. (4.3 ± 1.2), *p* = 0.98], and the same results were found in CDAI values [(22.9 ± 11.5) vs. (23.4 ± 10.7), *p* = 0.84]. No differences in the number of previous bDMARD treatments, the use of JAKis in monotherapy, serological status, sex or smoking habit were found between groups. Patients with higher BMI scores were significantly older and also had a longer disease duration; however, there was no significant correlation between these characteristics and leptin and adiponectin levels (see Table 1).

### 3.2. Association Between BMI and Serum Adipokines

**Figure 1:**
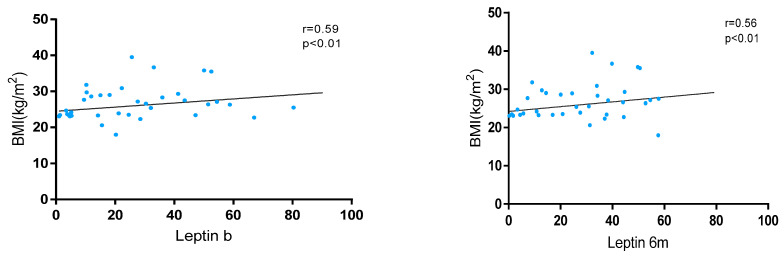
Correlation between BMI and leptin levels at baseline and 6 months after JAKi initiation.

Leptin levels showed a significant positive correlation with BMI at baseline (r = 0.59, *p* < 0.01) and at 6 months (r = 0.56, *p* < 0.01) in the whole cohort (Figure 1). These levels were significantly higher in patients with overweight/obesity than in normal weight at baseline [24.5 (11.5–42.4) vs. 18.1 (7.3–38.7); *p* < 0.01] and at 6 m [41.4 (26.9–57.2) vs. 34.2 (21.7–26.9); *p* < 0.01]. Also, variations in leptin levels were observed between baseline and 6 months without statistical significance [28.0 (13.6–47.2) vs. 32.1 (17.3–50.7); *p* = 0.052].

**Figure 2:**
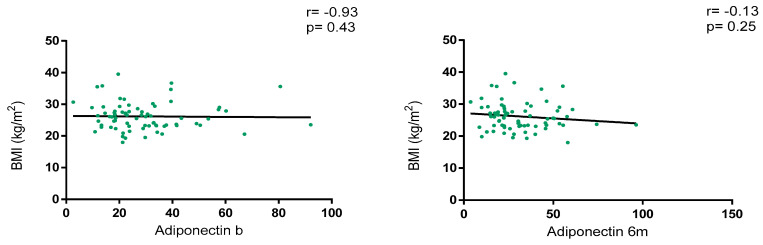
Correlation between BMI and adiponectin levels at baseline and 6 months after JAKi initiation.

Overall, adiponectin levels did not show a correlation with BMI at baseline (r = −0.93, *p* = 0.43) and at 6 m (r = −0.13, *p* = 0.25), as depicted in Figure 2. Regarding BMI classification, adiponectin levels were numerically higher in patients with normal weight vs. overweight/obesity at baseline [28.9 (18.5–36.5) vs. 23.2 (18.1–35.0); *p* = 0.46] and at 6 m [30.4 (20.6–42.9) vs. 23.0 (17.5–41.8); *p* = 0.30] without statistical significance. Variations in adiponectin levels were not significative between baseline and 6 m [23.8 (18.2–35.8) vs. 25.7 (18.6–41.7); *p* = 0.15].

A sub-analysis of adipokine levels according to the different grades of obesity was also performed and only significant differences were found between baseline and 6 m leptin levels in patients with overweight versus grade II obesity (Supplementary Material Table S1).

### 3.3. Association Between BMI and Disease Activity

**Figure 3:**
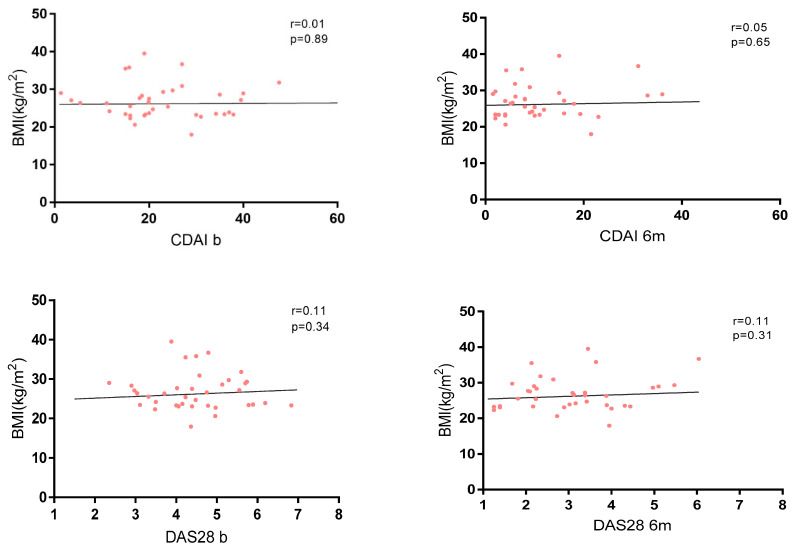
Correlation between BMI and disease activity measured by CDAI and DAS28.

No correlation between disease activity and BMI was found in the whole cohort at baseline and at 6 months (Figure 3), and no significant differences were found in terms of activity indexes and different grades of obesity (Supplementary Material Table S2).

In terms of treatment response, 50 patients (61.7%) achieved LDA measured by both the CDAI and DAS28 during the first six months of treatment, and there were no differences between both groups of BMI in achieving this outcome (Table 1).

### 3.4. Association Serum Adipokines and Treatment Response

After observing the absence of a correlation between BMI and serum adipokines, we analyzed the association of these parameters on treatment response in both groups.

**Figure 4:**
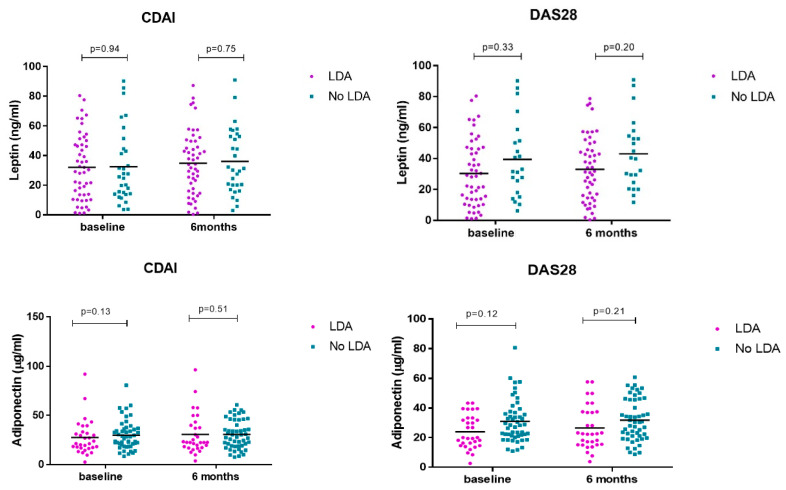
Serum leptin/adiponectin levels at baseline and 6 months divided according to response to treatment.

No differences in serum adipokine levels were observed at baseline and at 6 months in patients who achieved LDA vs. no-LDA measured by CDAI and DAS28 (Table 2 and Figure 4).

## 4. Discussion

Our results show that BMI do not influence the ability to achieve low disease activity in the first 6 months in patients with RA treated with JAKis, and this relationship is also observed in adipose tissue cytokine measurement.

Obesity is common in patients with RA, is associated with more severe symptoms and higher rates of disability among these patients, and can have a substantial impact on patient symptoms and outcomes [10]. Furthermore, the impact of BMI on the control of disease activity in rheumatic diseases has been widely reported in the literature. Indeed, several studies support the idea that obesity is a risk factor for poor response to treatment in RA, reducing the likelihood of achieving LDA in patients treated with cs/bDMARDs [11,12,13].

The mechanisms underlying the association between the impact of obesity and treatment response could be related to higher rates of chronic pain and opioid use, less reliable physical examination findings, and elevated inflammatory markers due to obesity’s negative impact triggering systemic inflammation. Adipose tissue has an endocrine function because it secretes many molecules (adipokines) involved in the inflammatory network and other pro-inflammatory cytokines, such as tumor necrosis factor-alpha (TNF-α), interleukin-1 (IL-1), monocyte chemotactic protein 1 (MCP-1) and interleukin-6 (IL-6) involved in RA pathogenesis. These inflammatory mediators may interfere with the mechanisms of action of these DMARDs, reducing their efficacy. Furthermore, obesity has been linked to altered drug pharmacokinetics, which may result in suboptimal drug concentrations in patients with a higher BMI, further impairing treatment response [6,14,15,16].

The impact of obesity as a comorbid condition in RA has been more extensively analyzed in the treatment with TNF inhibitors (TNFi), and there are currently numerous studies in which a higher BMI implies a worse response to bDMARDs, regardless of the route of administration [17,18,19]. In contrast, the inhibition of T cell co-stimulation, the blockade of B cells and IL-6 receptor blocking were not affected by obesity [19,20,21,22,23].

The results provided in our study are consistent with the available literature regarding the relationship between BMI and response rates to JAKi. These tsDMARDs have not shown reduced efficacy depending on BMI or weight according to data provided in clinical trials and post hoc studies [24,25,26,27]. Thus, BMI did not consistently affect tofacitinib, baricitinib and upadacitinib response, suggesting that these small molecules are effective oral treatment option regardless of baseline BMI, including patients with BMI ≥ 30 kg/m^2^ [24,25,26]. This conclusion is also reported with filgotinib, which did not lead to substantial changes in BMI, and BMI did not appear to affect the efficacy of this JAKi [27].

As previously mentioned, adipose tissue has endocrine functions and is capable of acting in the regulation of inflammatory mechanisms. Some studies have already analyzed the role of adipose tissue cytokines in the response to treatment in RA, especially TNFi and IL6 inhibitors (IL6i), and have found that IL6i can modulate these adipose tissue cytokines (especially leptin), while this modulation is not so evident in the case of TNFi drugs [19,28,29,30,31]. However, no studies focused on these pathophysiological mechanisms have been performed in patients treated with JAKi.

In our study, we observed a positive correlation between leptin levels and BMI; however, these parameters did not influence baseline disease activity or treatment response, suggesting that the mechanism of action of JAKi remains effective regardless of BMI status. This observation could be attributed to the pleiotropic role of leptin, which extends beyond energy balance regulation to promoting inflammation. In obesity, elevated leptin levels—resulting from increased adipose tissue—can enhance the secretion of pro-inflammatory cytokines, further activating the JAK/STAT pathway and amplifying inflammatory responses. Notably, the leptin receptor shares structural similarities with the signaling protein gp130, which exerts its biological function through JAK/STAT activation. This mechanism contributes to the chronic low-grade inflammation characteristic of obesity, a key driver of insulin resistance and other metabolic complications [6,21,22,23,24,25,26,27,28,29,30,31,32,33,34]. Consequently, JAK/STAT pathway inhibition may not only suppress inflammation but also potentially restore leptin sensitivity [35].

Conversely, adiponectin—an adipokine known for its anti-inflammatory properties—tends to be reduced in obesity, though its role in RA remains controversial [8]. In our analysis, adiponectin levels exhibited no significant variation and showed no association with disease activity or treatment response, including the achievement of low disease activity (LDA) or remission (REM). The existing literature suggests that adiponectin is less sensitive to treatment-induced changes, particularly with bDMARDs [19,36,37,38,39,40].

Our findings contribute to a deeper understanding of the pathophysiological mechanisms linking obesity and inflammation regulation in RA. Furthermore, this study is among the few to directly assess BMI, adipokine levels, and their potential influence on JAKi response. However, this study is not without limitations, mainly due to its relatively small sample size, something that did not allow us to perform a subtype analysis of different JAKis. Additionally, we did not measure other inflammatory cytokines implicated in both obesity and RA. So, further investigations are needed that should address this challenge.

## 5. Conclusions

In this study, we did not find association between obesity and the extent of LDA/REM in patients treated with JAKis. This may be due to the modulation of JAK/STAT signaling and its implications in the adipose tissue cytokine regulatory pathway. Therefore, this mechanism of action could be suitable for overweight/obese patients with RA.

## Figures and Tables

**Table 1:** Baseline clinical and demographic characteristics.

Variable	Total (*n* = 81)	BMI ≥ 25 kg/m^2^ (*n* = 44)	BMI < 25 kg/m^2^ (*n* = 37)	*p*-Value
Age mean (SD)	55.6 (11.6)	59.3 (10.6)	51.1 (11.1)	**0.01**
Sex (female) *n* (%)	70 (86.4)	36 (81.8)	34 (91.9)	0.16
Disease duration (years) mean (SD)	17.7 (10.2)	19.7 (10.2)	15.2 (9.7)	**0.04**
Smoking habit *n* (%)				0.08
Current	19 (23.5)	11 (25.0)	8 (21.6)	
Past	26 (32.1)	18 (40.9)	8 (21.6)	
Never	34 (42.0)	14 (31.8)	20 (54.1)	
JAKi				0.05
Baricitinib	37 (45.7)	16 (36.4)	21 (56.8)	
Filgotinib	17 (21.0)	8 (18.2)	9 (24.3)	
Tofacitinib	13 (16.0)	11 (25.0)	2 (5.4)	
Upadacitinib	14 (17.3)	9 (20.5)	5 (13.5)	
JAKi monotherapy	20 (24.7)	10 (22.7)	10 (27.0)	0.42
Use of concomitant methotrexate	34 (42.0)	18 (40.9)	16 (43.2)	0.50
bDMARD naïve *n* (%)	24 (29.6)	13 (29.5)	11 (29.7)	0.58
Number of previous bDMARDs	1 (0–3)	1 (0–3)	1 (0–2.5)	0.62
RF-positive (UI/mL) *n* (%)	70 (86.4)	37 (84.1)	33 (89.2)	0.36
ACPA-positive (UI/mL) *n* (%)	65 (80.2)	37 (84.1)	28 (75.7)	0.33
CRP b (mg/L) median (IQR)	3.5 (0.8–9.6)	4.4 (0.9–9.8)	2.9 (0.7–7.1)	0.76
DAS28 b mean (SD)	4.3 (1.1)	4.3 (1.0)	4.3 (1.2)	0.98
CDAI b mean (SD)	23.1 (11.1)	22.9 (11.5)	23.4 (10.7)	0.84
CRP 6 m (mg/L)	1.1 (0.5–4.5)	2.2 (0.5–6.0)	0.8 (0.5–4.1)	0.52
DAS28 6 m mean (SD)	2.8 (1.2)	3.0 (1.1)	2.9 (1.4)	0.65
CDAI 6 m mean (SD)	11.1 (9.5)	11.1 (8.8)	11.1 (10.5)	0.99
LDA-DAS28 *n* (%)	50 (61.7)	26 (59.1)	24 (64.9)	0.32
LDA-CDAI *n* (%)	50 (61.7)	29 (65.9)	21 (56.8)	0.26

BMI: body mass index; bDMARD: biologic disease-modifying antirheumatic drug; JAKi: Janus kinase inhibitor; RF: rheumatoid factor; ACPA: anti-citrullinated peptide antibody; CRP: C-reactive protein; DAS28: Disease Activity Score-28. CDAI: Clinical Disease Activity; LDA: low disease activity; SD: standard deviation; IQR: interquartile range.

**Table 2:** Adipokine levels regarding disease activity indexes.

Adipokine Levels	CDAI			DAS28		
	LDA	No-LDA	*p*-Value	LDA	No-LDA	*p*-Value
Leptin b (ng/mL)	29.7 (12.6–47.9)	25.6 (13.9–44.5)	0.94	28.2 (12.6–46.5)	27.6 (13.9–51.4)	0.33
Leptin 6 m (ng/mL)	34.1 (15.6–49.6)	31.2 (20.6–52.7)	0.75	31.4 (14.4–46.6)	39.7 (20.4–52.7)	0.20
Adiponectin b (μg/mL)	27.4 (19.8–36.3)	21.4 (16.8–33.3)	0.19	28.4 (21.2–37.1)	20.1 (13.9–31.8)	0.12
Adiponectin 6 m (μg/mL)	29.2 (19.2–45.6)	22.6 (17.3–39.9)	0.51	29.1 (18.9–45.8)	22.5 (15.5–37.5)	0.31

CDAI: Clinical Disease Activity Index; DAS28: Disease Activity Score-28; LDA: low disease activity; b: baseline; 6 m: 6 months. Data are presented in median and interquartile range.

## Data Availability

The data presented in this study are available on request from the corresponding author.

## Supplementary Materials

The following supporting information can be downloaded at: https://www.mdpi.com/article/10.3390/nu17050820/s1, Table S1: Leptin and adiponectin levels in patients depending on grade of obesity. Table S2: DAS28 and CDAI in patients depending on grade of obesity.

## References

[1] Swinburn B.A., Sacks G., Hall K.D., McPherson K., Finegood D.T., Moodie M.L., Gortmaker S.L. (2011). The global obesity pandemic: Shaped by global drivers and local environments. Lancet.

[2] Gremese E., Tolusso B., Gigante M.R., Ferraccioli C. (2014). Obesity as a risk and severity factor in rheumatic diseases (autoimmune chronic inflammatory diseases). Front. Immunol..

[3] Cildir G., Akıncılar S.C., Tergaonkar V. (2013). Chronic adipose tissue inflammation: All immune cells on the stage. Trends Mol. Med..

[4] Hauner H. (2005). Secretory factors from human adipose tissue and their functional role. Proc. Nutr. Soc..

[5] Maximus P.S., Al Achkar Z., Hamid P.F., Hasnain S.S., Peralta C.A. (2020). Adipocytokines: Are they the theory of everything?. Cytokine.

[6] Francisco V., Pino J., Gonzalez-Gay M.A., Mera A., Lago F., Gómez R., Mobasheri A., Gualillo O. (2018). Adipokines and inflammation: Is it a question of weight?. Br. J. Pharmacol..

[7] Francisco V., Pino J., Campos-Cabaleiro V., Ruiz-Fernandez C., Mera A., Gonzalez-Gay M.A., Gómez R., Gualillo O. (2018). Obesity, fat mass and immune system: Role for leptin. Front. Physiol..

[8] Fatel E.C.S., Rosa F.T., Simão A.N.C., Dichi I. (2018). Adipokines in rheumatoid arthritis. Adv. Rheumatol..

[9] Del Prete A., Salvi V., Sozzani S. (2014). Adipokines as potential biomarkers in rheumatoid arthritis. Mediat. Inflamm..

[10] Poudel D., George M.D., Baker J.F. (2020). The Impact of Obesity on Disease Activity and Treatment Response in Rheumatoid Arthritis. Curr. Rheumatol. Rep..

[11] Baker J.F., Reed G., Poudel D.R., Harrold L.R., Kremer J.R. (2022). Obesity and Response to Advanced Therapies in Rheumatoid Arthritis. Arthritis Care Res..

[12] Gialouri C.G., Pappa M., Evangelatos G., Nikiphorou E., Fragoulis G.E. (2023). Effect of body mass index on treatment response of biologic/targeted-synthetic DMARDs in patients with rheumatoid arthritis, psoriatic arthritis or axial spondyloarthritis. A systematic review. Autoimmun. Rev..

[13] Dey M., Zhao S.S., Moots R.J., Bergstra S.A., Landewe R.B., Goodson N.J. (2022). The association between increased body mass index and response to conventional synthetic disease-modifying anti-rheumatic drug treatment in rheumatoid arthritis: Results from the METEOR database. Rheumatology.

[14] Stavropoulos-Kalinoglou A., Metsios G.S., Koutedakis Y., Kitas G.D. (2011). Obesity in rheumatoid arthritis. Rheumatology.

[15] Braga G.C., Simões J.L.B., Teixeira Dos Santos Y.J., Menta-Filho J.C., Dulce Bagatini M. (2024). The impacts of obesity in rheumatoid arthritis and insights into therapeutic purinergic modulation. Int. Immunopharmacol..

[16] Lupoli R., Pizzicato P., Scalera A., Ambrosino P., Amato M., Peluso R., Di Minno M.N.D. (2016). Impact of body weight on the achievement of minimal disease activity in patients with rheumatic diseases: A systematic review and meta-analysis. Arthritis Res. Ther..

[17] Gremese E., Carletto A., Padovan M., Azteni F., Raffeiner B., Giardina A.R., Favalli E.G., Erre G.L., Gorla R., Galeazzi M. (2013). Gruppo Italiano di Studio sulle Early Arthritis (GISEA). Obesity and reduction of the response rate to anti-tumor necrosis factor α in rheumatoid arthritis: An approach to a personalized medicine. Arthritis Care Res..

[18] Klaasen R., Wijbrandts C.A., Gerlag D.M., Tak P.P. (2011). Body mass index and clinical response to infliximab in rheumatoid arthritis. Arthritis Rheum..

[19] Novella-Navarro M., Genre F., Hernández-Breijo B., Remuzgo-Martínez S., Martínez-Feito A., Peiteado D., Monjo I., Gonzalez-Gay M.A., Plasencia-Rodríguez C.H., Balsa A. (2022). Obesity and response to biological therapy in rheumatoid arthritis: The role of body mass index and adipose tissue cytokines. Clin. Exp. Rheumatol..

[20] Novella-Navarro M., Genre F., Martínez-Feito A., Pulito-Cueto V., Plasencia-Rodríguez C.H., Balsa A. (2023). Obesity and adipose tissue cytokines in rheumatoid arthritis treated with IL-6 inhibitors: Does the route of administration matter?. Clin. Exp. Rheumatol..

[21] Gardette A., Ottaviani S., Sellam J., Berenbaum F., Lioté F., Meyer A., Sibilia J., Fautrel B., Palazzo E., Dieudé P. (2016). Body mass index and response to tocilizumab in rheumatoid arthritis: A real life study. Clin. Rheumatol..

[22] D’Agostino M.A., Alten R., Mysler E., Le Bars M., Ye J., Murthy B., Heitzman J., Vadanici R., Ferracioli G. (2017). Body mass index and clinical response to intravenous or subcutaneous abatacept in patients with rheumatoid arthritis. Clin. Rheumatol..

[23] Ottaviani S., Gardette A., Roy C., Tubach F., Gill G., Palazzo E., Meyer O., Dieudé P. (2015). Body Mass Index and response to rituximab in rheumatoid arthritis. Jt. Bone Spine.

[24] Kremer J.M., Schiff M., Muram D., Zhong J., Alam J., Genovese M.C. (2018). Response to baricitinib therapy in patients with rheumatoid arthritis with inadequate response to csDMARDs as a function of baseline characteristics. RMD Open.

[25] Weinblatt M., Mysler E., Ostor A., Broadwell A., Jeka S., Dunlap K., Suboticki J., Enejosa J., Hendrickson B., Zhong S. (2020). Fri0140 Impact of Baseline Demographics and Disease Activity on Outcomes in Patients with Rheumatoid Arthritis Receiving Upadacitinib. Ann. Rheum. Dis..

[26] Dikranian A.H., Gonzalez-Gay M.A., Wellborne F., Alvaro-Gracia J.M., Takiya L., Stockert L., Paulissen J., Shi H., Tatulych S., Curtis J.R. (2022). Efficacy of tofacitinib in patients with rheumatoid arthritis stratified by baseline body mass index: An analysis of pooled data from phase 3 studies. RMD Open.

[27] Balsa A., Wassenberg S., Tanaka Y., Tournadre A., Orzechowski H.D., Rajendran V., Lendi U., Stiers P.J., Watson C., Caporali R. (2023). Effect of Filgotinib on Body Mass Index (BMI) and Effect of Baseline BMI on the Efficacy and Safety of Filgotinib in Rheumatoid Arthritis. Rheumatol. Ther..

[28] Corrado A., Colia R., Rotondo C., Sanpaolo E., Cantatore F.P. (2019). Changes in serum adipokines profile and insulin resistance in patients with rheumatoid arthritis treated with anti-TNF-α. Curr. Med. Res. Opin..

[29] Gonzalez-Gay M.A., Garcia-Unzueta M.T., Berja A., Gonzalez-Juanatey C., Miranda-Filloy J.A., Vazquez-Rodriguez T.R., De Matias M.J., Martin J., Dessein P.H., Llorca J. (2009). Anti-TNF-alpha therapy does not modulate leptin in patients with severe rheumatoid arthritis. Clin. Exp. Rheumatol..

[30] Pulito-Cueto V., Remuzgo-Martínez S., Genre F., Calvo-Alen J., Aurrecoechea E., Llorente I., Triguero-Martinez A., Blanco R., Llorca J., Ruiz-Lucea E. (2020). Anti-IL-6 therapy reduces leptin serum levels in patients with rheumatoid arthritis. Clin. Exp. Rheumatol..

[31] Hoffman E., Rahat M.A., Feld J., Elias M., Rosner I., Kaly S., Lavie I., Gazitt T., Zisman D. (2019). Effects of Tocilizumab, an Anti-Interleukin-6 Receptor Antibody, on Serum Lipid and Adipokine Levels in Patients with Rheumatoid Arthritis. Int. J. Mol. Sci..

[32] Wang T., He C. (2018). Pro-inflammatory cytokines: The link between obesity and osteoarthritis. Cytokine Growth Factor Rev..

[33] Frühbeck G. (2006). Intracellular signalling pathways activated by leptin. Biochem. J..

[34] Ghantous C.M., Azrak A., Hanache S., Abou-Kheir W., Zeidan A. (2015). Differential Role of Leptin and Adiponectin in Cardiovascular System. Int. J. Endocrinol..

[35] Dodington D.W., Desai H.R., Woo M. (2018). JAK/STAT—Emerging Players in Metabolism. Trends Endocrinol. Metab..

[36] Pulito-Cueto V., Remuzgo-Martínez S., Genre F., Calvo-Alen J., Aurrecoechea E., Llorente I., Triguero-Martinez A., Blanco R., Llorca J., Ruiz-Lucea E. (2022). Role of adiponectin in non-diabetic patients with rheumatoid arthritis undergoing anti-IL-6 therapy. Clin. Exp. Rheumatol..

[37] Fioravanti A., Tenti S., Bacarelli M.R., Damiani A., Li Gobbi F., Bandinelli F., Cheleschi S., Galeazzi M., Benucci M. (2019). Tocilizumab modulates serum levels of adiponectin and chemerin in patients with rheumatoid arthritis: Potential cardiovascular protective role of IL-6 inhibition. Clin. Exp. Rheumatol..

[38] Virone A., Bastard J.P., Fellahi S., Capeau J., Rouanet S., Sibilia J., Ravaud P., Berenbaum F., Gottenberg J.E., Sellam J. (2019). Comparative effect of tumour necrosis factor inhibitors versus other biological agents on cardiovascular risk-associated biomarkers in patients with rheumatoid arthritis. RMD Open.

[39] Baker J.F., Odell J.R., England B.R., Giles J.T., Newcomb J.A., George M.D., Thiele G., Morelan L., Bridges S.L., Curtis J.R. (2024). Lower body mass and lower adiposity are associated with differential responses to two treatment strategies for rheumatoid arthritis. Ann. Rheum. Dis..

[40] Giardullo L., Corrado A., Maruotti N., Cici D., Mansueto N., Cantatore F.P. (2021). Adipokine role in physiopathology of inflammatory and degenerative musculoskeletal diseases. Int. J. Immunopathol. Pharmacol..

