# Supplementary material for: Do Obesity and Adipose Tissue Cytokines Influence the Response to Janus Kinase Inhibitors in Rheumatoid Arthritis?
Source: Nutrients. 2025 Feb 27;17(5):820. doi: 10.3390/nu17050820 (PMC11901994; doi:10.3390/nu17050820)
Supplement: Supplementary file 1 [file nutrients-17-00820-s001.zip › nutrients-3486209-supplementary.pdf]

Suplementary Material Table S1: leptin and adiponectin levels in patients depending on grade of obesity

	Overweight (n=33)	Grade I obesity (n=6)	Grade II obesity (n=5)	p-value
Leptin b	32.3 (14.7-47.5)	39.3 (24.5-80.7)	67.3 (55.1-67.0)	0.02*
Leptin 6m	37.0 (20.3-57.2)	38.6 (30.5-59.9)	72.0 (52.7-80.2)	0.04*
Adiponectin b	23.2 (18.2-30.9)	27.2 (15.8-39.4)	19.5 (12.6-60.1)	0.93
Adiponectin 6m	22.8 (18.0-39.4)	28.2 (10.3-44.0)	23.3 (16.9-41.7)	0.90

**statistical significance obtained by Kruskal-Wallis test. Post-hoc analysis using Dunnet test revealed significant differences in leptin b and leptin 6m between overweight and Grade II obesity.*

Supplementary Material Table S2: DAS28 and CDAI in patients depending on grade of obesity

	Overweight (n=33)	Grade I obesity (n=6)	Grade II obesity (n=5)	p-value
DAS28 b	4.3 (1.1)	5.0 (0.6)	4.2 (0.3)	0.24
DAS28 6m	3.0 (1.1)	2.7 (1.0)	3.4 (1.5)	0.93
CDAI b	23.2 (12.5)	28.2 (11.8)	19.1 (10.7)	0.20
CDAI 6m	11.1 (9.2)	9.8 (7.0)	12.8 (10.4)	0.53
